# An effect of inhibitory load in children while keeping working memory load constant

**DOI:** 10.3389/fpsyg.2014.00213

**Published:** 2014-03-14

**Authors:** Andy Wright, Adele Diamond

**Affiliations:** Department of Psychiatry, University of British ColumbiaVancouver, BC, Canada

**Keywords:** executive function, inhibitory control, self-regulation, cognitive control, executive control, spatial Stroop task, Simon task, stimulus-response compatibility

## Abstract

Children are slower and more error-prone when the correct response is counter to their initial inclination (incongruent trials) than when they just need to do what comes naturally (congruent trials). Children are almost always tested on a congruent-trial block and then on an incongruent-trial block. That order of testing makes it impossible to determine whether worse performance on incongruent trials is due to the need to inhibit a pre-potent response, the need to clear the rule for Block 1 from working memory, some other demand of task-switching, or some combination of these. However, if the congruent block and incongruent blocks each have only one rule (e.g., “press on the same side as the stimulus” for congruent trials and “press on the side opposite the stimulus” for incongruent trials, as on the hearts and flowers task) and children’s performance when the incongruent block is presented *first* is fully comparable to their performance when it is presented second, the only possible explanation for their worse performance on incongruent versus congruent trials would seem to be the added inhibitory demand on incongruent trials. Certainly, worse performance on Block 1 would not be due to inefficient clearing of working memory or task-switching demands. We tested 96 children (49 girls) 6–10 years of age on the hearts and flowers test with order of congruent and incongruent blocks counterbalanced across children. Children were slower and made more errors on incongruent trials regardless of task order. We expected task-switching demands to account for some of the variance, but to our surprise, performance was fully comparable on the incongruent block whether it came first or second. These results indicate that increasing inhibitory demands alone is sufficient to impair children’s performance in the face of no change in working memory demands, suggesting that inhibition is a separate mental function from working memory.

## INTRODUCTION

It is hotly debated whether working memory and inhibitory controls are separable or not. Many argue that working memory is all that is required; no need to posit a separate inhibitory control ability (Cohen et al., 2002; Egner and Hirsch, 2005; Nieuwenhuis and Yeung, 2005; Hanania and Smith, 2010; Munakata et al., 2011; Chatham et al., 2012). Others posit that inhibitory control is an ability in its own right, separate from working memory (e.g., Gernsbacher, 1993; Levy and Anderson, 2002; Gazzaley et al., 2005; Leroux et al., 2006; Diamond, 2009; Zanto and Gazzaley, 2009).

When performing tasks that require working memory and inhibitory control, children are slower and make more errors on incongruent (incompatible) blocks than on congruent (compatible) ones. Each block may have only one rule but incongruent blocks add an inhibitory demand. When the incongruent block follows a congruent one, poorer performance on the incongruent block could easily be due to problems in efficiently clearing the congruent rule from working memory. Thus the working memory demand might be greater on Block 2 than on Block 1. However, when the incongruent block is presented first, worse performance on the incongruent block compared to the congruent one should be attributable to the greater inhibitory demand in the incongruent block. Such performance, if found, would seem to provide evidence in favor of working memory and inhibitory control being separable. To our knowledge, the study reported here is the first to present the incongruent-trial block before the congruent one to children.

For this study we wanted a task (a) that requires working memory (not just memory maintenance or short-term memory), (b) where the congruent and incongruent blocks each present only one rule to hold and manipulate in working memory, and (c) where there is clear empirical evidence that incongruent trials require a response counter to subjects’ first inclination or response tendency [i.e., that “response inhibition,” a component of “inhibitory control” (Diamond, 2013) is required]. The hearts and flowers task fit that bill.

The hearts and flowers task (previously called the dots task; Davidson et al., 2006; Diamond et al., 2007) is a hybrid combining elements of Simon and spatial Stroop tasks. For congruent trials, subjects are to obey the rule, “Press on the same side as the stimulus.” For incongruent trials, subjects are to follow the rule, “Press on the side opposite the stimulus.” Both of those blocks require working memory because we do not have “same side” or “opposite side” hands (we have right and left hands); on each trial those rules must be translated into which hand to use (requiring that subjects mentally work with the rule they are holding in mind). This is an important difference between Simon tasks and the hearts and flowers task. Simon tasks require short-term memory, but not working memory, because they require simply holding two rules in mind (“For Stimulus A, press on the right” and “For Stimulus B, press on the left”), not mentally manipulating that information in any way.

Short-term memory involves only “memory maintenance,” only holding information in mind (as required by a forward digit span task where you need to repeat back information you just heard in the order in which you heard it). Working memory, in contrast, requires memory maintenance plus working with the information you are holding in mind (as would be required if you need to repeat back information you just heard re-ordering it according to size, numerical or alphabetical order, or some other criterion; Baddeley, 1992; Petrides, 1994, 1995; D’Esposito et al., 1995, 1998; Owen et al., 1996; Cohen et al., 1997; Smith and Jonides, 1999; Smith et al., 1998).

**FIGURE 1:**
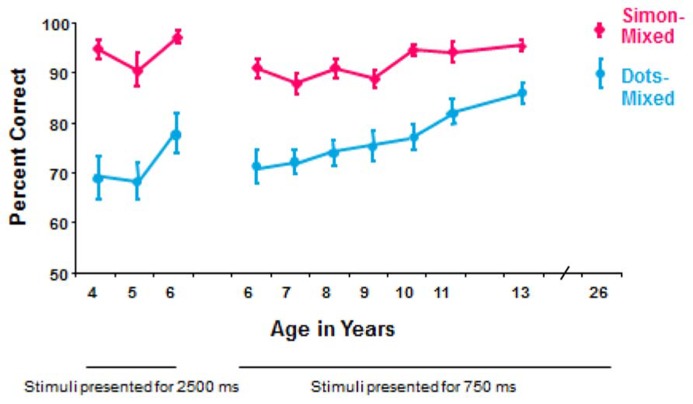
**Comparison of performance on the mixed conditions of the hearts and flowers task with dot stimuli and of a Simon task.** This is based on within-subject comparisons of 314 participants (roughly 30 per age; equal numbers of males and females) tested on both tasks using the same equipment and same timing parameters (Davidson et al., 2006). At every age, participants were significantly faster and significantly more accurate on the Simon task. The dot stimuli were a gray disk and a black-and-white-striped disk; that is the only difference between the older dots version of the task and the current hearts and flowers task. The rules for the Simon task were, “If you see a butterfly, press the button on the left, whether the butterfly appears on the left or right. If you see a frog, press the button on to the right, whether the frog appears on the left or right.”

Children at all ages that were tested (4–13 years) and young adults perform significantly better (fewer errors and faster responding) on the Simon task (with the memory demand of only holding information in mind) than on the hearts and flowers task [with the memory demands of holding information in mind plus manipulating that information (translating “same side” and “opposite side” into “right hand” or “left hand”)]; see **Figure 1**.

People have a pre-potent tendency to respond toward a stimulus (Fitts and Seeger, 1953; Simon and Rudell, 1967; Lu and Proctor, 1995; Kornblum et al., 1999; Hommel et al., 2004; Hommel, 2011). That must be inhibited when the stimulus and its associated response are on opposite sides (incongruent trials). Adults and children are slower and make more errors when the stimulus appears on the side opposite its associated response than when stimulus appears on the same side as its associated response (called the Simon effect, the spatial incompatibility effect, or stimulus-response incompatibility; *adults*: Lu and Proctor, 1995; Kornblum et al., 1999; Kunde and Stocker, 2002; Hommel et al., 2004; Hommel, 2011; *children*: Gerardi-Coulton, 2000; Davidson et al., 2006; Mullane et al., 2009). Indeed, when monkeys are to respond away from a visual stimulus, the neuronal population vector in primary motor cortex (coding the direction of planned movement) initially points toward the stimulus and only then shifts to the required direction (showing a pre-potent tendency at the neuronal level to respond toward a stimulus; to do otherwise requires that that impulse be inhibited; Georgopoulos et al., 1989; Georgopoulos, 1994). This has been seen in humans using lateralized motor-readiness evoked potentials (Valle-Inclan, 1996) and event-related optical imaging (EROS; DeSoto et al., 2001). DeSoto et al. (2001) showed that incongruent trials elicit simultaneous activation of both motor cortices (necessitating the need for one to be inhibited) whereas congruent trials elicit brain activity in only the motor cortex associated with the response.

Thus, the hearts and flowers task met all three of our criteria. In the standard hearts and flowers task, participants are instructed (a) to press the response button on the same side (left or right) as the stimulus (a red heart) on Block 1 (the congruent block), (b) to press the response button on the side opposite the stimulus (a red flower) on Block 2 (the incongruent block), and (c) to flexibly switch between those two rules on Block 3 where the stimulus might be a heart or flower (the mixed block). Participants of every age that has been tested (4–13 years, plus young adults) are slower and make more errors on the mixed block (Davidson et al., 2006). Young adults, however, are as fast and accurate on the incongruent block as they are on the congruent one. In contrast, children of all ages tested (4–13 years) are slower and make more errors on the incongruent block than the congruent one (Davidson et al., 2006).

The hearts and flowers task has been used to demonstrate executive function gains from the *Tools of the Mind* preschool curriculum (Diamond et al., 2007), to provide the first demonstration in children of a difference in executive function performance by COMT genotype (Diamond et al., 2004), and to demonstrate a sex difference in which version of the COMT gene is more beneficial for executive functions (Evans et al., 2009). It has been shown to accurately assess executive functions in both typically developing children and children with Down syndrome (Edgin et al., 2010). Zaitchik et al. (2013) found that the hearts and flowers task, but not several other tasks in their executive function battery, predicted their composite measure of vitalist biology as it is constructed by children (as predicted) controlling for age and IQ. The relation between hearts and flowers performance and on-the-face-of-it task-demands on their biology measures also held up (e.g., inhibitory control as indexed by hearts and flowers predicted animism judgments more strongly than purely factual knowledge about bodily function).

Using the hearts and flowers task, the present study tested two competing hypotheses:

(1) Children might err on the incongruent block because of the addition of an inhibitory demand – the need to resist responding on the same side as the stimulus, responding on the opposite side instead. For the congruent block children need only do what comes naturally, but for the incongruent block they must inhibit that and do the opposite. Thus, Hypothesis 1 is that children make more errors and take more time to respond on the incongruent block because of their immature ability to exercise inhibitory control.
(2) Perhaps, however, it is the task-switching requirement (and need to efficiently delete the rule for Block 1 from working memory when performing Block 2) that gives children difficulty. The incongruent block routinely follows the congruent one on most tasks, including the hearts and flowers task. Hypothesis 2 is that it is the difficulty of switching from the rule to always press on the same side as the stimulus to the rule to always pressing on the side opposite the stimulus that accounts for children’s slower response times and increased errors on the incongruent block. We know that switching from one rule to another can be difficult even for adults, and especially for children (Hartman and Hasher, 1991; Allport and Wylie, 2000; Monsell and Driver, 2000; Cepeda et al., 2001; Zelazo et al., 2003; Crone et al., 2006; Yeung et al., 2006).

It may be that children do not wipe their mental slate clean when they begin Block 2, and so are still holding the now-irrelevant rule from Block 1 in mind. That would mean that the memory load for them on Block 2 would be greater because they would be holding in mind both the congruent and incongruent rules. If that is the case, then reversing the order in which the congruent and incongruent blocks are presented should get rid of poorer performance on the incongruent block. Hypothesis 1, on the other hand, leads to the prediction that reversing the order would do nothing to diminish the gap in children’s performance on Blocks 1 and 2 (they would still be slower and less accurate on the incongruent block, even if it came first, because the inhibitory-control demand would be the same).

In a between-subjects design we tested half the children at each age with the congruent block first and half with the incongruent block first on the hearts and flowers task.

## MATERIALS AND METHODS

### PARTICIPANTS

Data were obtained from 96 children, ranging in age from 6 to 10 years (49% male, 51% female; see **Table 1**), from public elementary schools throughout the Lower Mainland of BC, Canada. Participants were recruited through their schools and 95% were tested at their school. The other five children were tested at our child development lab at the University of British Columbia.

**Table 1:** Number of participants within each age and gender group.

Age group (years)	Mean age (years)	SD	*N*	Gender		Location of testing	
				Female (%)	Male (%)	Our lab	School
6	6.50	0.31	18	50	50	2	16
7	7.64	0.24	15	47	53	0	15
8	8.50	0.34	16	56	44	1	15
9	9.49	0.29	23	43	57	2	21
10	10.35	0.23	24	54	46	0	24
Totals			96	51	49	5	91

The majority of participants who provided ethnic information were Caucasian of European descent (52%), 16% were of East Asian descent (most were Chinese), 12% were of South Asian descent (most were Indian), and the rest were of other ethnic backgrounds. All were fluent in English. Informed consent was obtained from a parents of each child, and informed assent was obtained from each child, before testing. All participants received a small present for their participation.

### PROCEDURE

Within each age × gender grouping, half the participants were randomly assigned to get the congruent block first and half to get the incongruent block first. Participants were tested individually in a quiet room while wearing noise cancelation headphones. The stimuli were presented on a Dell 43 cm touchscreen monitor attached to an IBM ThinkPad Lenovo T6 laptop computer. The hearts and flowers task was administered using Presentation^®^ software.

**FIGURE 2:**
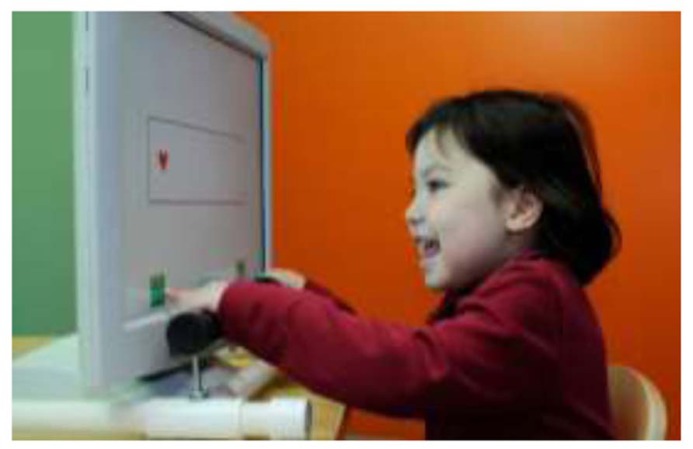
**A child performing the hearts and flowers test using a touchscreen monitor and handlebars**.

Participants held a handlebar with both hands to keep the distance from their hands to the response buttons constant. They were instructed to use only their pointer finger to press the response button on the screen (see **Figure 2**).

All participants completed a button practice task before moving onto hearts and flowers. Two response buttons appeared on the touchscreen monitor for the practice task. Children were to press a response button as soon as they saw a smiley face appear on it. This task provided baseline choice-reaction time data as well as serving to acclimate children to using the handlebars and to pressing the left and right response buttons on screen. Children were corrected if they reached across the midline to respond. They were also corrected if they left their finger on the monitor after their response, did not keep their hands on the handlebars before the smiley face appeared, or did not replace their finger on the handlebars after pushing the button.

The same procedure for the hearts and flowers task was used as previously reported (Davidson et al., 2006; Diamond et al., 2007). On each trial, a red heart or a red flower appeared on either the left or right side of the screen. A correct response to the heart was to press the response box on the touchscreen monitor on the same side as the heart. A correct response to the flower was to press the response box on the side opposite the flower.

On each trial, a horizontal rectangle (6 cm × 18 cm) was presented in the center of the screen. An orienting crosshair was presented for 500 ms at center fixation at the outset of each trial, and then disappeared, replaced 500 ms later by a stimulus on the left or right. One stimulus was presented per trial. The stimulus was presented for 750 ms to children ≥7 years of age and for 1500 ms to children 6 years of age. [These timing parameters had been determined to be age appropriate by Davidson et al. (2006)]. Each test block was preceded by instructions and a demonstration of the task followed by a practice block. Understanding of the rule was demonstrated by getting at least three of the four trials correct in the practice block. If understanding was not demonstrated on the first practice block, the child was instructed again and given another practice block (two children in the incongruent-first condition and two in the congruent-first condition needed a second practice block). No participant in the study failed to pass practice. The congruent and incongruent test blocks consisted of 12 trials each. There were 33 trials in the mixed block. Trials in each block were presented in the same pseudo-random order to each child.

## RESULTS

The two dependent measures were speed [reaction time (RT)] and accuracy [percentage of correct responses]. Trials with RTs faster than 250 ms were excluded for being too fast to have been in response to the stimulus (resulted in 5 trials being excluded). RTs 2 standard deviations above or below a subject’s mean were also excluded from analyses for being outliers (3 trials excluded). Percentage of correct responses was calculated by dividing the number of correct responses by the total number of responses (excluding the exceptions just mentioned). Only correct trials were used in calculating a child’s mean RT in each test block.

### RESULTS FOR SPEED OF RESPONDING

No significant difference was found between RTs during button practice (our baseline measure of choice RT) of children who received the incongruent block first and children who received the congruent block first [ANCOVA: *F*(1,89) = 0.83, ns] controlling for age, gender, and ethnicity. That is, there was no difference in baseline speed between children who received one order of presentation or the other. Choice RT did not vary by gender [*F*(1,89) = 0.053, ns] or ethnicity [*F*(3,89) = 0.21, ns]. Older children of course had faster choice RTs than younger children (all subjects: *F*(1,78) = 12.34, *p* < 0.001; only those receiving the same timing parameters [excludes 6-year-olds (who were given longer to respond)]: *F*(1,71) = 4.17, *p* < 0.05).

Excluding the 6-year-olds, who were given much more time to respond, RTs declined significantly over age for only the mixed condition of the hearts and flowers task [mixed block: *F*(3,74) = 2.639, *p* < 0.05; congruent block: *F*(3,74) = 0.69, ns; incongruent block: *F*(3,74) = 0.05, ns]. Since age is a continuous variable, we also examined this using multiple regression (controlling for gender and ethnicity) and received comparable results [mixed block: *F*(1,72) = 5.07, *p* < 0.03; congruent block: *F*(1,72) = 0.70, ns; incongruent block: *F*(1,76) = 0.02, ns]. RTs did not differ for any block by gender or ethnicity.

**FIGURE 3:**
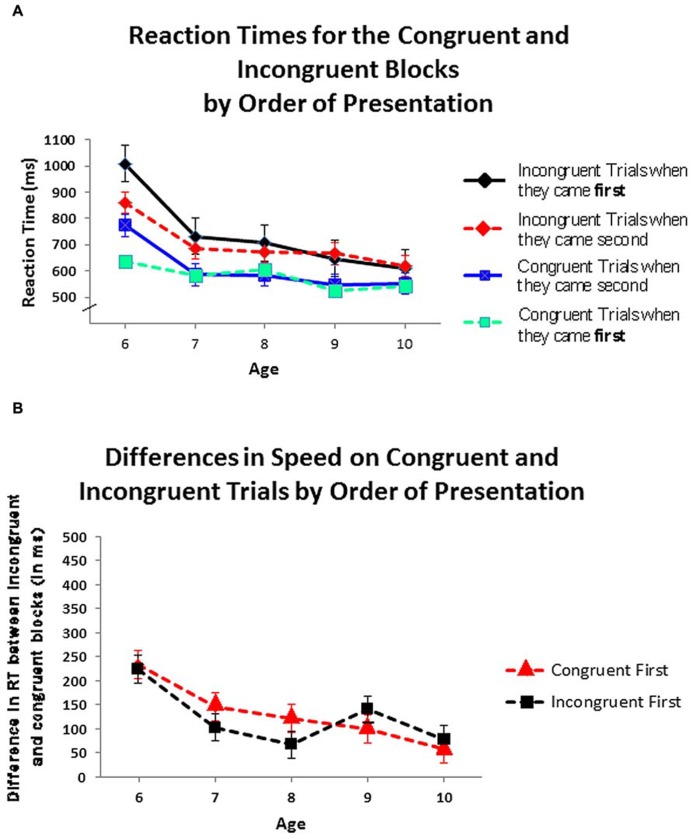
**(A)** Speed of responding on the congruent and incongruent blocks by order of presentation. **(B)** Differences in speed on incongruent and congruent trial blocks by order of presentation.Values greater than zero, as all are, indicate that children responded faster on congruent than on incongruent blocks.

To compare the differences in RT between the congruent block and incongruent block for each order of testing, an ANOVA was conducted with order in which the congruent and incongruent blocks were presented as between-subject factors and block type (congruent, incongruent) as a within-subject factor; age, gender, and ethnicity were not included given the absence of any significant effects for those variables or their interactions. For the congruent-first condition (the order usually used for the hearts and flowers task) RTs in the congruent block (Mean = 596.44 ms, SD = 116.81) were significantly faster than in the incongruent block (Mean = 725.17 ms, SD = 167.17): *F*(1,45) = 25.79, *p* < 0.001. For the incongruent-first condition, RTs in the congruent block (Mean = 600.79 ms, SD = 138.05) were also significantly faster than in the incongruent block (Mean = 721.37 ms, SD = 180.89): *F*(1,47) = 19.02, *p* < 0.001. In both orders of testing, *at every age*, children responded faster in the congruent block than in the incongruent one (see **Figure 3A**). The difference between RTs on incongruent and congruent blocks did not differ by the order in which the blocks were presented: *F*(1,93) = 1.41, ns. *At no age* did the within-child difference in speed on the two blocks differ significantly by order of presentation (see **Figure 3B**). All of the above also held for each gender and for each ethnic group analyzed separately.

**FIGURE 4:**
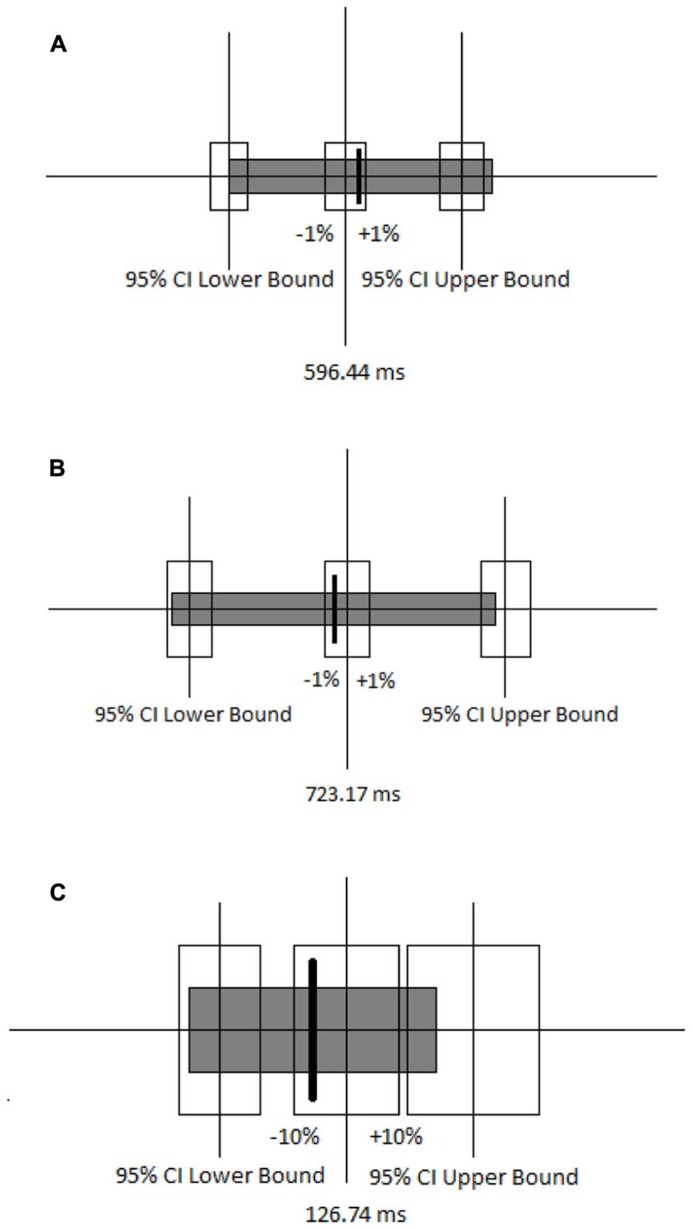
**(A) Ninety-five percent confidence interval around the mean RT for the congruent block when it came first is shown by the unfilled boxes.** The thick line and gray box indicate the mean and 95% confidence interval for congruent trials when they came second. **(B)** Ninety-five percent confidence interval around the mean RT for the incongruent block when it came first is shown by the unfilled boxes. The thick line and gray box indicate the mean and 95% confidence interval for incongruent trials when they came second. **(C)** Ninety-five percent confidence intervals around the mean RT for the differences between the congruent and incongruent blocks when the congruent block came first is shown by the unfilled boxes. The thick line and gray box indicate the mean and 95% confidence interval for the difference between the congruent and incongruent blocks when the incongruent block came first.

An insignificant *p*-value is not always sufficient for concluding that two conditions are equivalent (Lesaffre, 2008). Equivalence between the congruent-first and incongruent-first conditions on both congruent and incongruent trials was tested by setting a 95% confidence interval around the mean RT for each block in the congruent-first condition, and specifying equivalence as the RTs in the incongruent-first condition being within plus or minus 1%. The mean RTs on the congruent block (**Figure 4A**) and incongruent block (**Figure 4B**) in the incongruent-first condition fell within the specified interval of equivalence when compared with the mean RT on the corresponding blocks in the congruent-first condition. This means that the mean RTs were equivalent for congruent trials whether they came first or second and the mean RTs were also equivalent for incongruent trials regardless of the order in which they we presented. The distribution of RTs was also similar. The equivalence of the difference in RT between the congruent and incongruent blocks in both congruent-first and incongruent-first conditions was also tested using the 95% confidence interval (**Figure 4C**). Equivalence here was defined as being within plus or minus 10% the difference [note that the difference RTs is far smaller than actual RTs, so 10% of a difference is miniscule (roughly 12 ms or so)].

### RESULTS FOR ACCURACY OF RESPONDING

Because accuracy data are binary at the individual trial level, a generalized estimating equation using a binary logistic equation was used to compare the difference in accuracy between the first two trial blocks in the congruent-first condition and the incongruent-first condition. Accuracy did not differ for any block by ethnicity. Children >7 years made no errors on the button practice that preceded testing on hearts and flowers.

Accuracy improved over age from 6 to 10 years on both the congruent and incongruent blocks (chi square: congruent block: χ^2^(1, *N* = 96) = 4.13, *p* < 0.04, odds ratio = 1.64; incongruent block: χ^2^(1, *N* = 96) = 7.11, *p* < 0.01, odds ratio = 1.39). Excluding 6-year-olds (who were given more time to respond than all other children) accuracy improved over age only on the mixed block [χ^2^(1, *N* = 77) = 7.18, *p* < 0.01]. All children >7 years were correct on all trials in the congruent block. Accuracy did not differ by ethnicity on any block or by gender on the incongruent or mixed blocks. However, girls were correct on more trials than boys in the congruent block (all ages: χ^2^(1, *N* = 96) = 5.70, *p* < 0.02, odds ratio = 2.26; only children 7–10 years old: χ^2^(1, *N* = 77) = 9.40, *p* < 0.02, odds ratio = 3.54).

**FIGURE 5:**
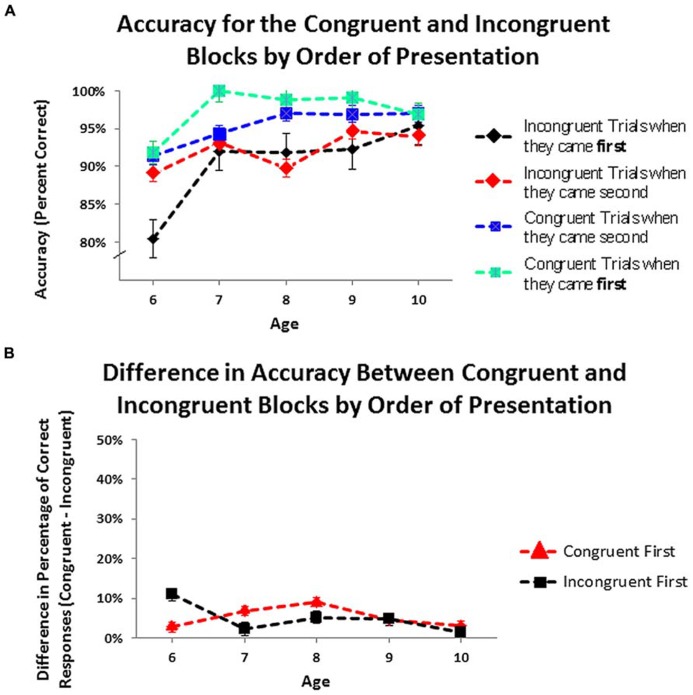
**(A)** Accuracy on the congruent and incongruent blocks by order of presentation. **(B)** Difference in accuracy between congruent and incongruent blocks by order of presentation.Values greater than zero indicate that children were correct on more trialsin congruent than in incongruent blocks.

In the congruent-first condition, participants responded more accurately on the congruent trial block (mean = 97.26%, SD = 5.07%) than on the incongruent one (mean = 92.17%, SD = 8.50%): χ^2^(1, *N* = 46) = 7.50, *p* < 0.006, odds ratio = 4.58. In the incongruent-first condition as well, the percentage of correct responses was higher in the congruent block (mean = 95.50%, SD = 6.77%) than in the incongruent one (mean = 91.00%, SD = 7.96%): χ^2^(1, *N* = 50) = 8.23, *p* < 0.004, odds ratio = 3.02. *At every age*, regardless of the order in which the conditions were tested, children made fewer errors in the congruent block than in the incongruent one (see **Figure 5A**).

An ANOVA within a General Linear Model with age as a continuous between-subject variable, with order of trial blocks and gender as categorical between-subject variables, and with block type (congruent or incongruent) as a categorical within-subject factor was conducted to determine whether the difference in accuracy between the two blocks of trials was similar or different in the two orders of testing. The difference in accuracy between congruent and incongruent blocks did not vary by order of presentation [*F*(1,89) = 2.04, ns]. *At no age* did the within-child difference in accuracy on the two blocks differ significantly by order of presentation (see **Figure 5B**). All of the above also held regardless of ethnicity or gender and there were no significant effects of, or interactions with, gender or ethnicity. Children of 6 years were as accurate on the incongruent block as the congruent one, so including them in the analyses showed a significant increase in the difference in percentage of correct trials on congruent versus incongruent trials by age [*F*(1,89) = 5.29, *p* < 0.02]. Including only the children who received the same timing parameters (children 7–10 years), there was no change in this difference over age.

**FIGURE 6:**
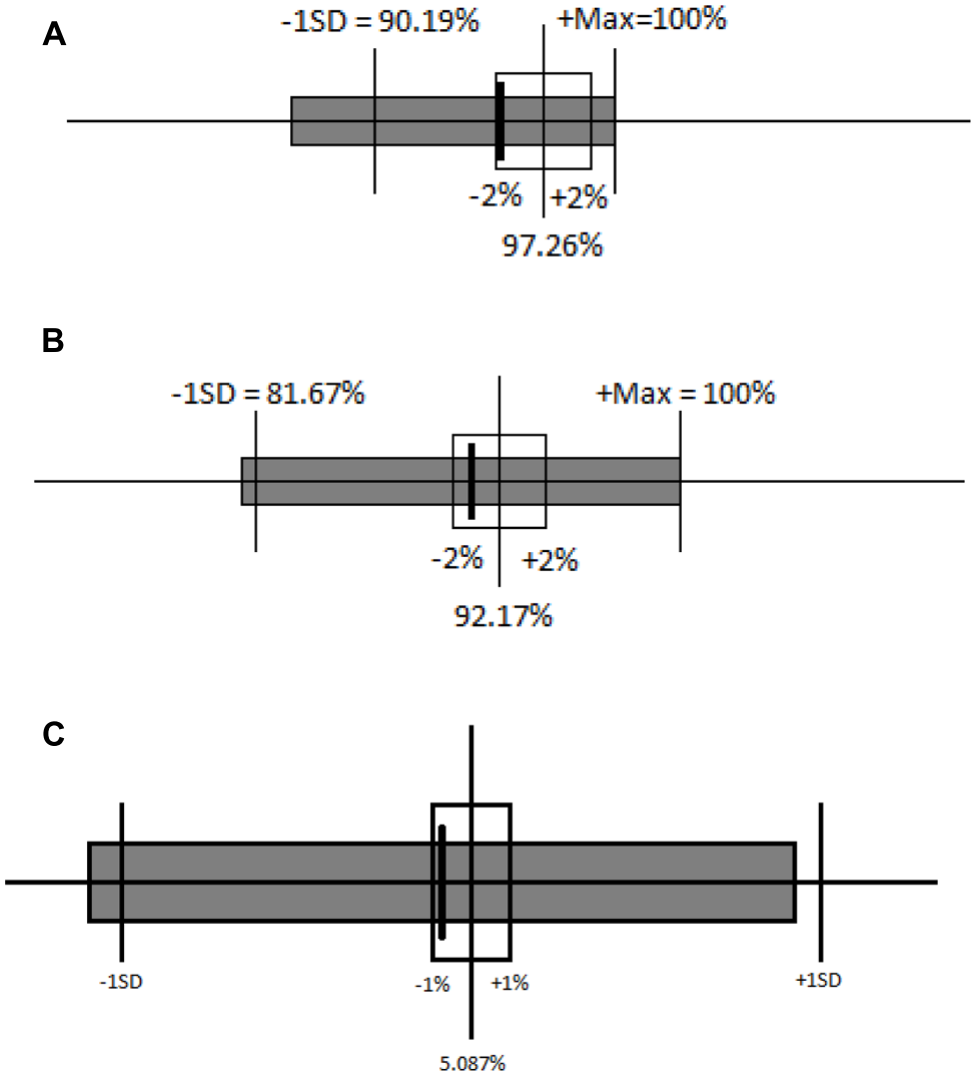
**(A)** Ninety-five percent confidence interval around the mean accuracy for the congruent block in the congruent-first condition (unfilled boxes and solid lines) and comparison to the incongruent-first condition (solid box), plus or minus 2%. **(B)** Ninety-five percent confidence interval around the mean accuracy for the incongruent block in the congruent-first condition (unfilled boxes and solid lines) and comparison to the incongruent-first condition (solid box), plus or minus 2%. **(C)** Ninety-five percent confidence interval around the mean difference in accuracy between congruent and incongruent blocks in congruent-first and incongruent-first conditions. Solid lines represent the accuracy difference in the congruent-first condition and the gray box represents the accuracy difference in the incongruent-first condition.

Again, a difference that fails to reach significance is insufficient to demonstrate equivalence, so a specified interval of equivalence was again used. The interval was set at 2% because one incorrect answer causes a large change in accuracy values. Both mean accuracy on the congruent block (**Figure 6A**) and on the incongruent block (**Figure 6B**) for the incongruent-first order of testing fell within the interval of equivalence for the congruent-first order of testing. The distribution of percentage of correct responses on incongruent blocks was also equivalent across the two orders of testing (see **Figure 6A**). The distributions of percentage of correct responses on congruent blocks, however, did differ: Children made more errors on congruent trials when they followed incongruent ones than when congruent trials came first, providing a hint of a subtle difference in performance on congruent trials by order of testing. The equivalence of the difference in accuracy between the congruent and incongruent blocks in both congruent-first and incongruent-first conditions was also tested using the 95% confidence interval (**Figure 6C**). This interval of equivalence was set at 1% because a difference score always has a small range of variability. The difference in accuracy between the two blocks was equivalent regardless of which block came first.

## DISCUSSION

This study explored what the critical difference is between incongruent and congruent blocks that accounts for why children perform so much worse on incongruent blocks. For the first time we know of, the order in which congruent and incongruent blocks were presented to children was varied. Worse performance on the incongruent block of the hearts and flowers task when it comes second could be accounted for by greater working memory demands (subjects might still be holding the first rule in mind when performing Block 2), greater inhibitory demands, task-switching demands, or some combination of those. However, worse performance on the incongruent block when it comes first (as found here) can be accounted for only by greater demands on inhibition.

Regardless of the order in which the congruent and incongruent blocks were presented, children at every age were slower and made more errors on the incongruent block than the congruent one. That is, they performed worse on the incongruent block even when it was presented first, and this difference in performance was no greater when the incongruent block came second. These results strongly support that the source of the difficulty for children is *not* switching from the rule in Block 1 to the rule in Block 2, nor does the source of their problem seem to be holding in mind the rule for Block 1 when they perform Block 2 (not having efficiently deleted it from working memory); the source of their difficulty seems to be the need to inhibit a pre-potent response on incongruent trials. These results also show that varying the demand on inhibition (the incongruent block requires inhibition of a pre-potent behavioral tendency whereas the congruent block does not) holding working memory demands constant (when the congruent block is presented first it requires holding one rule in mind and when the incongruent block is presented first it, too, requires holding only one rule in mind) is sufficient to produce a decrement in children’s performance evident both in poorer speed and accuracy.

A memory theorist might protest that worse performance on incongruent trials even when they come first could result from the difficulty of maintaining the rule sufficiently active in working memory for it to “win” in the battle for controlling behavior in the face of interference from the natural inclination to press on the same side as a stimulus. This seems to allow for no disproof of a working memory hypothesis, however, because it asserts that whenever the demand on inhibition is increased (whenever a strong disposition to act a certain way must be suppressed or overridden) ipso facto the demand on working memory is increased. What we know about the incongruent condition is that a strong competing response is present (a strong tendency to give a response that would be incorrect must not win; it must be inhibited). We also know that the incongruent and congruent conditions require holding and manipulating in working memory only one rule each. Those are objective behavioral observations. It is an unproven hypothesis that inhibition of a competing response is accomplished by working memory “working harder.” It is also an unproven hypothesis that inhibition of a competing response is accomplished by executive attention working harder to keep one’s attention focused on the relevant rule. This paper reports what is behaviorally available for observation.

These results provide evidence of the consequences of a greater inhibitory demand (on incongruent trials), independent of any difference in the quantity or complexity of what must be held in working memory. In the face of no change in the working memory demand, increasing the demand on inhibitory control alone is sufficient to induce more errors and slower responding in children. Adults may not appreciate how inordinately difficult inhibition is for young children because it is so much less difficult for adults (adults show no difference in performance on congruent and incongruent blocks of the hearts and flowers task, or usually of Simon or spatial Stroop tasks, showing errors and slower responding only on mixed blocks [Lu and Proctor, 1995]). Often conditions differ in both working memory and inhibitory control demands making it impossible to attribute differences in performance specifically to working memory or inhibitory control. Here, where demands on working memory and inhibitory have been dissociated, it is possible to see that increasing inhibitory control demands alone is sufficient to induce worse performance in children 6–10 years of age.

## Conflict of Interest Statement

The authors declare that the research was conducted in the absence of any commercial or financial relationships that could be construed as a potential conflict of interest.

## References

[B1] Allport A. Wylie G. (2000). “Task switching, stimulus-response bindings, and negative priming,” in *Control of cognitive processes: Attention and Performance XVII* eds Monsell S. Driver J. (Cambridge, MA: MIT Press) 35–70

[B2] Baddeley A. (1992). Working memory. *Science* 255 556–559 10.1126/science.1736359 1736359

[B3] Cepeda N. J. Kramer A. F. Gonzalez D. S. (2001). Changes in executive control across the life span: examination of task-switching performanc. *Dev. Psychol.* 37 715–730 10.1037/0012-1649.37.5.715 11552766

[B4] Chatham C. H. Claus E. D. Kim A. Curran T. Banich M. T. Munakata Y. (2012). Cognitive control reflects context monitoring, not motoric stopping in response inhibition. *PLoS ONE *7:2. 10.1371/journal.pone.0031546 PMC3288048 22384038

[B5] Cohen J. D. Braver T. S. Brown J. W. (2002). Computational perspectives on dopamine function in prefrontal cortex. *Curr. Opin. Neurobiol.* 12 223–229 10.1016/S0959-4388(02)00314-8 12015241

[B6] Cohen J. D. Perlstein W. M. Braver T. S. Nystrom L. E. Noll D. C. Jonides J. (1997). Temporal dynamics of brain activation during a working memory task. *Nature* 386 604–607 10.1038/386604a0 9121583

[B7] Crone E. A. Bunge S. A Van Der Molen M. W. (2006). Switching between tasks and responses: a developmental study. *Dev. Sci.* 9 278–287 10.1111/j.1467-7687.2006.00490.x 16669798

[B8] Davidson M. C. Amso A. Anderson L. C. Diamond A. (2006). Development of cognitive control and executive functions from 4 to 13 year: evidence from manipulations of memory, inhibition, and task switching. *Neuropsychologia* 44 2037–2078 10.1016/j.neuropsychologia.2006.02.006 16580701 PMC1513793

[B9] DeSoto M. C. Fabiani M. Geary D. C. Gratton G. (2001). When in doubt, do it both ways: brain evidence of the simultaneous activation of conflicting motor responses in a spatial Stroop task. *J. Cogn. Neurosci.* 13 523–536 10.1162/08989290152001934 11388924

[B10] D’Esposito M. Aguirre G. K. Zarahn E. Ballard D. Shin R. K. Lease J. (1998). Functional MRI studies of spatial and nonspatial working memory. *Cogn. Brain Res.* 7 1–13 10.1016/S0926-6410(98)00004-4 9714705

[B11] D’Esposito M. Detre J. A. Alsop D. C. Shin R. K. Atlas S. Grossman M. (1995). The neural basis of the central executive system of working memory. *Nature* 378 279–281 10.1038/378279a0 7477346

[B12] Diamond A. (2009). When in competition against engrained habits, is conscious representation sufficient or is inhibition of the habit also needed? *Dev. Sci.* 12 20–22 10.1111/j.1467-7687.2008.00773.x 19120407 PMC2621328

[B13] Diamond A. (2013). Executive functions. *Annu. Rev. Psychol.* 64 135–168 10.1146/annurev-psych-113011-143750 23020641 PMC4084861

[B14] Diamond A. Barnett S. Thomas J. Munro S. (2007). Preschool program improves cognitive control. *Science* 318 1387–1388 10.1126/science.1151148 18048670 PMC2174918

[B15] Diamond A. Briand L. Fossella J. Gehlbach L. (2004). Genetic and neurochemical modulation of prefrontal cognitive functions in children. *Am. J. Psychiatry* 161 125–132 10.1176/appi.ajp.161.1.125 14702260

[B16] Edgin J. O. Mason G. M. Allman M. J. Capone G. T. DeLeon I. Maslen C. (2010). Development and validation of the Arizona Cognitive Test Battery for Down syndrome. *J. Neurodev. Disord.* 2 149–164 10.1007/s11689-010-9054-3 21274406 PMC3026140

[B17] Egner T. Hirsch J. (2005). Cognitive control mechanisms resolve conflict through cortical amplification of task-relevant information. *Nat. Neurosci.* 8 1784–1790 10.1038/nn1594 16286928

[B18] Evans J. W. Fossella J. Hampson E. Kirschbaum C. Diamond A. (2009). Gender differences in the cognitive functions sensitive to the level of dopamine in prefrontal cortex. *Poster presentation, Inaugural Conference on “Executive Function and Dysfunction,” (Jan. 15) University of Boulder, Boulder, CO and at the Association for Psychological Science Annual Meeting (May 25)* San Francisco, CA

[B19] Fitts P. M. Seeger C. M. (1953). S-R compatibility: spatial characteristics of stimulus and response codes. *J. Exp. Psychol.* 81 174–176 10.1037/h0062827 13084867

[B20] Gazzaley A. Cooney J. W. Rissman J D’Esposito M. (2005). Top-down suppression deficit underlies working memory impairment in normal aging. *Nat. Neurosci.* 8 1298–1300 10.1038/nn1543 16158065

[B21] Georgopoulos A. (1994). Population activity in the control of movement. *Int. Rev. Neurobiol.* 37 103–119 7883475 10.1016/s0074-7742(08)60241-x

[B22] Georgopoulos A. P. Lurito J. T. Petrides M. Schwartz A. B. Massey J. T. (1989). Mental rotation of the neuronal population vector. *Science* 243 234–236 10.1126/science.2911737 2911737

[B23] Gerardi-Coulton G. (2000). Sensitivity to spatial conflict and the development of self-regulation in children 24-36 months of age. *Dev. Sci.* 3 397–404 10.1111/1467-7687.00134

[B24] Gernsbacher M. A. (1993). Less skilled readers have less efficient suppression mechanisms. *Psychol. Sci.* 4 294–298 10.1111/j.1467-9280.1993.tb00567.x PMC4191741 25309046

[B25] Hanania R. Smith L. B. (2010). Selective attention and attention switching: towards a unified developmental approach. *Dev. Sci.* 13 622–635 10.1111/j.1467-7687.2009.00921.x 20590726 PMC2939469

[B26] Hartman M. Hasher L. (1991). Aging and suppression: memory for previously relevant information. *Psychol. Aging* 6 587–594 10.1037/0882-7974.6.4.587 1777147

[B27] Hommel B. (2011). The Simon effect as tool and heuristic. *Acta Psychol.* 136 189–202 10.1016/j.actpsy.2010.04.011 20507830

[B28] Hommel B. Proctor R. W Vu K.-P. L. (2004). A feature integration account of sequential effects in the Simon task. *Psychol. Res.* 68 1–17 10.1007/s00426-003-0132-y 14752663

[B29] Kornblum S. Stevens G. T. Whipple A. Requin J. (1999). The effects of irrelevant stimuli: 1. The time course of S-S and S-R consistency effects with Stroop-like stimuli, Simon-like tasks, and their factorial combinations. *J. Exp. Psychol.* 25(A) 688–714 10.1037/0096-1523.25.3.688

[B30] Kunde W. Stocker C. (2002). A Simon effect for stimulus-response duration. *Q. J. Exp. Psychol.* 55A 581–592 10.1080/02724980143000433 12047060

[B31] Leroux G. Joliot M. Dubal S. Mazoyer B. Tzourio-Mazoyer N. Houde O. (2006). Cognitive inhibition of number/length interference in a Piaget-like task in young adults: evidence from ERPs and fMRI. *Hum. Brain Mapp.* 27 498–509 10.1016/j.clinph.2009.06.003 16161161 PMC6871484

[B32] Lesaffre E. (2008). Superiority, equivalence, and non-inferiority trials. *Bull. NYU Hosp. Jt. Dis.* 2 150–154 18537788

[B33] Levy B. J. Anderson M. C. (2002). Inhibitory processes and the control of memory retrieval. *Trends Cogn. Sci. (Regul. Ed.)* 6 299–305 10.1016/S1364-6613(02)01923-X 12110363

[B34] Lu C. H. Proctor R. W. (1995). The influence of irrelevant location information on performance: a review of the Simon and spatial Stroop effects. *Psychon. Bull. Rev.* 2 174–207 10.3758/BF03210959 24203654

[B35] Monsell S. Driver J. (eds). (2000). *Control of Cognitive Processes: Attention and Performance XVIII*. Cambridge, MA: MIT Press

[B36] Mullane J. C. Corkum P. V. Klein R. M. McLaughlin E. (2009). Interference control in children with and without ADHD: a systematic review of Flanker and Simon Task Performance. *Child Neuropsychol.* 15 321–342 10.1080/09297040802348028 18850349

[B37] Munakata Y. Herd S. A. Chatham C. H. Depue B. E. Banich M. T O’Reilly R. C. (2011). A unified framework for inhibitory control. *Trends Cogn. Sci. (Regul. Ed.)* 15 453–459 10.1016/j.tics.2011.07.011 21889391 PMC3189388

[B38] Nieuwenhuis S. Yeung N. (2005). Neural mechanisms of attention and control: losing our inhibitions? *Nat. Neurosci.* 8 1631–1633 10.1038/nn1205-1631 16306886

[B39] Owen A. M. Evans A. C. Petrides M. (1996). Evidence for a two-stage model of spatial working memory processing within lateral frontal cortex: a positron emission tomography study. *Cereb. Cortex* 6 31–38 10.1093/cercor/6.1.31 8670636

[B40] Petrides M. (1994). “Frontal lobes and working memory: evidence from investigations of the effects of cortical excisions in nonhuman primates,” in *Handbook of Neuropsychology*, Vol. 9 eds Boller F. Grafman J. (Amsterdam: Elsevier Science Publishers) 59–82

[B41] Petrides M. (1995). Functional organization of the human frontal cortex for mnemonic processing: evidence from neuroimaging studies. *Ann. N. Y. Acad. Sci.* 769 85–96 10.1111/j.1749-6632.1995.tb38133.x 8595046

[B42] Simon R. J. Rudell A. P. (1967). Auditory S-R compatibility: the effect of an irrelevant cue on information processing. *J. Appl. Psychol.* 51 300–304 10.1037/h0020586 6045637

[B43] Smith E. E. Jonides J. (1999). Storage and executive processes in the frontal lobes. *Science* 283 1657–1661 10.1126/science.283.5408.1657 10073923

[B44] Smith E. E. Jonides J. Marshuetz C. Koeppe R. A. (1998). Components of verbal working memory: evidence from neuroimaging. *Proc. Natl. Acad. Sci. U.S.A.* 95 876–882 10.1073/pnas.95.3.876 9448254 PMC33811

[B45] Valle-Inclan F. (1996). The Simon effect and its reversal studied with event-related potentials. *Int. J. Psychophysiol.* 23 41–53 10.1016/0167-8760(96)00027-X 8880365

[B46] Yeung N. Nystrom L. E. Aronson J. A. Cohen J. D. (2006). Between-task competition and cognitive control in task switching. *J. Neurosci.* 26 1429–1438 10.1523/JNEUROSCI.3109-05.2006 16452666 PMC6675498

[B47] Zanto T. P. Gazzaley A. (2009). Neural suppression of irrelevant information underlies optimal working memory performance. *J. Neurosci.* 29 3059–3066 10.1523/JNEUROSCI.4621-08.2009 19279242 PMC2704557

[B48] Zaitchik D. Iqbal Y. Carey S. (2013). The effect of executive function on biological reasoning in young children: an individual differences study. *Child Dev.* 85 160–175 10.1111/cdev.1214 23889035

[B49] Zelazo P. D. Muller U. Frye D. Marcovitch S. (2003). The development of executive function in early childhood. *Monogr. Soc. Res. Child Dev.* 68 137 10.1111/j.0037-976X.2003.00269.x 14723273

